# Aging, cardiorespiratory fitness and sympathetic transduction

**DOI:** 10.18632/aging.204091

**Published:** 2022-05-16

**Authors:** Myles W. O’Brien, Said Mekary, Derek S. Kimmerly

**Affiliations:** 1 Division of Kinesiology, School of Health and Human Performance, Faculty of Health, Dalhousie University, Halifax, Nova Scotia, Canada; 2 Department of Family Medicine, Université de Sherbrooke, Sherbrooke, Québec, Canada

**Keywords:** neurovascular transduction, aerobic fitness, adrenergic receptor, muscle sympathetic nerve activity, exercise training

Advancing age lowers maximal aerobic fitness and increases cardiovascular disease risk (e.g., hypertension). Engagement in sufficient moderate-to-vigorous physical activity to maintain a high cardiorespiratory fitness (CRF) attenuates the negative cardiovascular impact of aging. The vasoconstrictor arm of the sympathetic nervous system is critical for effective short-term arterial blood pressure regulation. Sympathetic neural activity directed towards skeletal muscle resistance vessels (muscle sympathetic nerve activity; MSNA) causes peripheral vasoconstriction, which is exaggerated in older adults [1]. Signal averaging techniques have been developed [2], and refined [3], to quantify the communication between MSNA and the corresponding pressor or local vascular responses (i.e., sympathetic transduction). The assessment of sympathetic transduction may help uncover the divergent cardiovascular implications of inactive (lower CRF) versus active (higher CRF) aging.

Using microneurographic recordings of spontaneous common peroneal MSNA and non-invasive beat-by-beat arterial pressure (via finger photoplethysmography), our laboratory demonstrated that lower CRF (peak oxygen consumption) was associated with greater sympathetic transduction and larger blood pressure variability responses in older adults [4]. The inverse CRF–blood pressure variability relationship remained after controlling for potential confounders (i.e., MSNA burst frequency, sympathetic transduction, age, sex). Higher blood pressure variability, with larger systolic blood pressure surges, has clinical relevance as it contributes to the development of adverse cardiovascular events [5]. Accordingly, our observations supported that CRF level in older adults was inversely related to the magnitude of sympathetic transduction and provided insight into the sympathetic regulation of arterial pressure.

While MSNA burst frequency and incidence increase with age [1], older adults exhibit lower sympathetic transduction responses than their younger counterparts [3]. This observation remained even if younger and older males were matched for either CRF (relative peak oxygen consumption) or age- and sex-specific normative CRF percentiles [6]. Considering that advancing age and higher CRF exhibit opposing effects on cardiovascular health and disease risk, it may seem somewhat counterintuitive that both factors were inversely associated with sympathetic transduction. Clearly, these conflicting observations highlight that having higher or lower sympathetic transduction cannot be overtly labelled as either ‘good’ or ‘bad’ with respect to sympathetic vascular function. While often studied in isolation, we position that a better understanding of the (dis)similar mechanisms involved in the neural regulation of vascular function between age and CRF are required.

Insight into the mechanistic underpinnings of sympathetic transduction have been gleaned using intra-arterial infusions of phentolamine and phentolamine + angiotensin II (to compensate for phentolamine-induced decreases in basal blood flow) have demonstrated that the vasoconstrictor responses to spontaneous bursts of MSNA are mediated via α-adrenergic receptors [7]. Older adults exhibit attenuated α-adrenergic receptor-mediated vasoconstrictor responses compared to younger counterparts, which may be due to reduced sensitivity and/or saturation of post-junctional α_1_- and α_2_-receptors. Similarly, aerobic training (15 m/min at 15% incline, 60 min/day, 5 days/week for 10–12 weeks) attenuated α-adrenergic receptor-mediated vasoconstriction in older male Fischer rats [8]. Accordingly, alterations at the α-adrenergic receptor level, including attenuated vasoconstrictor responses that accompany both age and aerobic fitness is a non-specific (α_1_- versus α_2_-receptors) mechanism common to both factors.

Post-ganglionic sympathetic nerves terminate at the vascular adventitia-media border of arteries and resistance vessels. As indicate above, the pressor responses following bursts of MSNA are due to contraction of the vascular smooth muscle cells (i.e., vasoconstriction) located within the *tunica media*. The vascular remodelling that occurs with increasing age and aerobic training may each contribute to sympathetic transduction. However, age and aerobic training exhibit divergent effects on arterial compliance and wall-to-lumen ratio (↓ with aging: ↑ with training/CRF), suggesting that this not an overlapping mechanism contributing to attenuated sympathetic transduction.

The magnitude of sympathetic neural vasoconstrictor responses may also be attenuated by locally released endothelial-derived vasodilator substances (i.e., nitric oxide, endothelial-derived hyperpolarizing factors, prostaglandins). Older age and lower CRF are both associated with decreased endothelial-dependent vasodilation, primarily characterized by impaired conduit artery flow-mediated dilation responses. The nadir arterial pressure observed following cardiac cycles absents of MSNA bursts (i.e., sympathetic quiescence) may provide insight into the independent impact of these opposing vasodilatory pathways on sympathetic transduction. However, these nadir pressure responses to non-burst cardiac cycles were unrelated to CRF or blood pressure variability in older adults [4]. Furthermore, when matched for CRF, younger and older males exhibited similar nadir arterial pressure responses to non-burst sequences. It is unclear whether the contribution of specific endothelial-derived vasodilators during sympathetic quiescence are altered with aging and/or aerobic training status. However, our observations suggest that they likely play a minor role regarding the similar effects of age and CRF on lower sympathetic transduction responses [4].

In conclusion, there remains much to be understood regarding the independent associations of aging versus CRF on attenuated sympathetic neural control of peripheral vasoconstrictor and pressor responses. Longitudinal aging and aerobic training interventions are warranted, with current evidence pointing towards a primary impact on mechanisms involved with reduced α-adrenergic receptor-mediated vasoconstriction. The impact of aging and CRF on sympathetic neural co-transmission (e.g., neuropeptide Y and adenosine triphosphate) and subsequent transduction outcomes are another avenue for future exploration. Understanding the divergent and overlapping mechanisms of age versus CRF on sympathetic transduction may provide insight into the development of optimal interventions for the treatment of age-related cardiovascular conditions characterized by excessive sympathetic outflow.
